# Supplementary material for: Enhanced Efficiency and Stability of Perovskite Solar Cells Through Neodymium-Doped Upconversion Nanoparticles with TiO2 Coating
Source: Molecules. 2025 May 14;30(10):2166. doi: 10.3390/molecules30102166 (PMC12114041; doi:10.3390/molecules30102166)
Supplement: Supplementary file 1 [file molecules-30-02166-s001.zip › molecules-3581334-supplementary.pdf]

Supporting Information

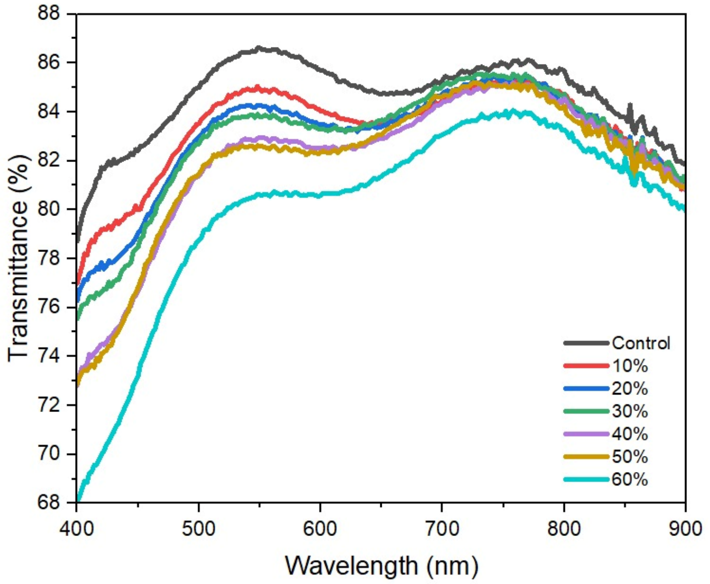

Figure S1. Transmittance spectra of incorporating varying weight ratios of UCNPs@TiO_2 in the mesoporous layer.

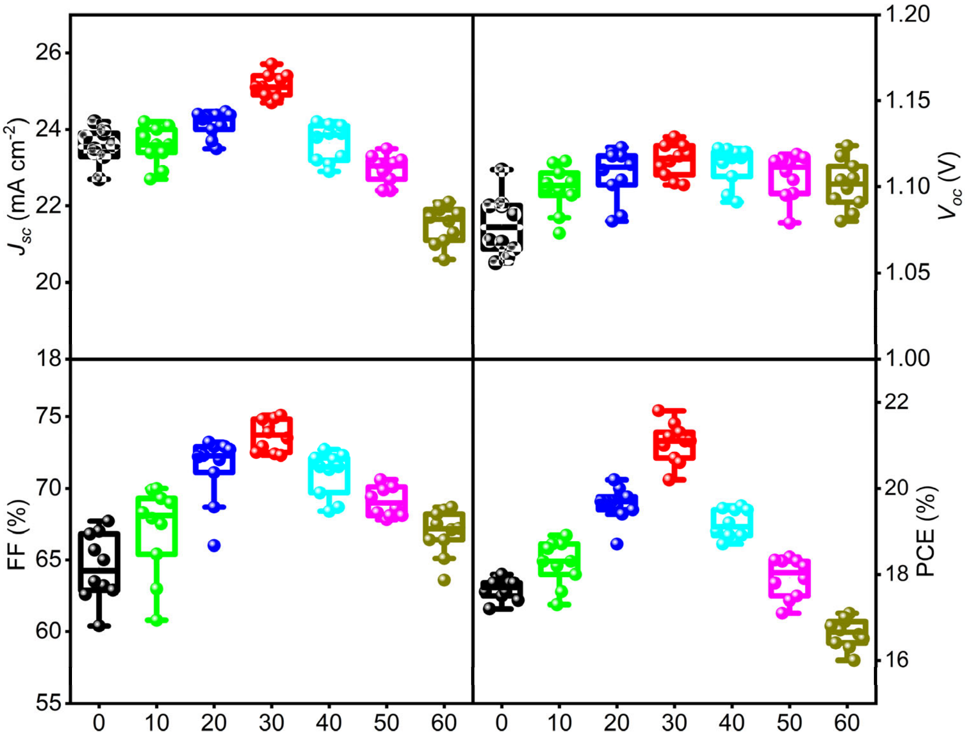

Figure S2. Statistical data of photovoltaic performance analysis of PSC devices incorporating varying concentrations of UCNPs@TiO_2 in the ETL (ten devices per condition).

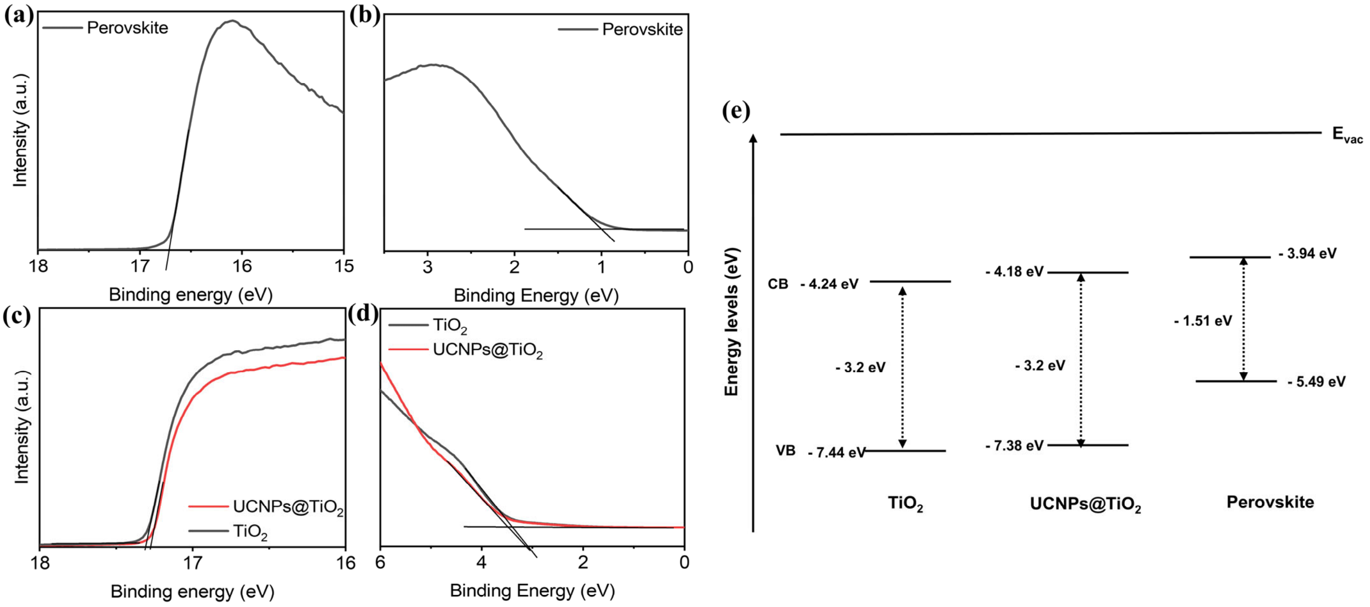

Figure S3. UPS characterization of TiO_2 and perovskite layers, comparing samples with and without 30% UCNPs: (**a**,**c**) Secondary electron cut-off spectra of TiO_2 and perovskite layers; (**b**,**d**) Corresponding valence band spectra of TiO_2 and perovskite layers. (**e**) energy level allignment of samples with and without 30% UCNPs.
